# Radiological features of screening-detected and interval breast cancers and subsequent survival in Eastern Finnish women

**DOI:** 10.1038/s41598-024-60740-0

**Published:** 2024-05-01

**Authors:** Aki Nykänen, Mazen Sudah, Amro Masarwah, Ritva Vanninen, Hidemi Okuma

**Affiliations:** 1 Department of Clinical Radiology, Diagnostic Imaging Centre, Kuopio University Hospital, Puijonlaaksontie 2, 70210 Kuopio, Finland; 2 School of Medicine, Institute of Clinical Medicine, University of Eastern Finland, Yliopistonranta 1, 70210 Kuopio, Finland; 3 Cancer Center of Eastern Finland, University of Eastern Finland, Yliopistonranta 1, 70210 Kuopio, Finland

**Keywords:** Breast cancer, Cancer imaging

## Abstract

Interval breast cancers are diagnosed between scheduled screenings and differ in many respects from screening-detected cancers. Studies comparing the survival of patients with interval and screening-detected cancers have reported differing results. The aim of this study was to investigate the radiological and histopathological features and growth rates of screening-detected and interval breast cancers and subsequent survival. This retrospective study included 942 female patients aged 50–69 years with breast cancers treated and followed-up at Kuopio University Hospital between January 2010 and December 2016. The screening-detected and interval cancers were classified as true, minimal-signs, missed, or occult. The radiological features were assessed on mammograms by one of two specialist breast radiologists with over 15 years of experience. A χ^2^ test was used to examine the association between radiological and pathological variables; an unpaired *t* test was used to compare the growth rates of missed and minimal-signs cancers; and the Kaplan–Meier estimator was used to examine survival after screening-detected and interval cancers. Sixty occult cancers were excluded, so a total of 882 women (mean age 60.4 ± 5.5 years) were included, in whom 581 had screening-detected cancers and 301 interval cancers. Disease-specific survival, overall survival and disease-free survival were all worse after interval cancer than after screening-detected cancer (*p* < 0.001), with a mean follow-up period of 8.2 years. There were no statistically significant differences in survival between the subgroups of screening-detected or interval cancers. Missed interval cancers had faster growth rates (0.47% ± 0.77%/day) than missed screening-detected cancers (0.21% ± 0.11%/day). Most cancers (77.2%) occurred in low-density breasts (< 25%). The most common lesion types were masses (73.9%) and calcifications (13.4%), whereas distortions (1.8%) and asymmetries (1.7%) were the least common. Survival was worse after interval cancers than after screening-detected cancers, attributed to their more-aggressive histopathological characteristics, more nodal and distant metastases, and faster growth rates.

## Introduction

Mammography is the standard method used to screen for breast cancer in order to reduce breast cancer mortality through the earlier detection of a malignancy, although a considerable proportion of breast cancers can be missed with mammography^1^. Breast cancers that are diagnosed between scheduled screenings after a negative screening result are referred to as “interval breast cancers”. Interval cancers are an important indicator of the quality of a breast-cancer-screening program^2^. Breast cancers can be classified based on the findings of previous mammographic examinations: cancers that were not visible on previous screening mammograms are considered “true” cancers; “minimal-signs” cancers showed nonspecific minor abnormalities on previous screening mammograms that did not lead to a diagnosis of breast cancer; cancers that were visible on a previous screening mammogram but were overlooked are “missed” cancers; and cancers that were not mammographically visible at diagnosis are “occult” cancers^3^.

The recognized risk factors for interval breast cancers are high breast density, younger age, premenopausal status, lower body mass index (BMI), current or previous use of hormone replacement therapy, family history of breast cancer, and previous false-positive mammography examination^1,4,5^. The interval breast cancer rates for biennial screening programs range from 8.4 to 21.1 per 10,000 screenings, and for the majority of biennial screening studies, interval breast cancers represent 17–30% of the cancers that occur in screening participants^5^.

As many as 30% of breast cancers may be missed with screening mammography, and up to 50% of interval and screening-detected breast cancers present visible findings on previous screening mammograms^6–13^. Review methods affect the proportion of interval cancers classified as “missed”, which partly explains the marked variation in the rates of missed cancers reported in different studies^14^. Several recognized technological and human-related factors can lead to breast cancer being missed on mammography. Lesions may be missed because of poor image quality or poor positioning. Architectural distortion, lesions obscured by dense parenchyma, and diffusely infiltrating processes may make lesions difficult to recognize, so they are not perceived as abnormalities. Benign-appearing nodules, slowly developing asymmetries, and the absence of substantial growth over time can give the false impression of a benign etiology, and thus lead to the incorrect interpretation of an abnormality^15,16^. Other factors to be considered are information overload and perception errors that occur when viewing several projections of mammograms simultaneously without adequate image enlargement.

Survival after interval breast cancer and the difference in the survival of patients with screening-detected and interval cancers have been studied in the last few decades, but with differing results. Therefore, a survival assessment of patients who have received modern treatments in recent years is required to assess the significance of interval cancers today^3,17–35^.

Therefore, the aim of this study was to evaluate and classify interval cancers, assess their radiological and histopathological features and growth rates of both screening-detected and interval breast cancers and the subsequent survival rates in an Eastern Finnish population.

## Methods

In accordance with the Finnish national regulations, ethics committee approval is not mandatory in retrospective registry studies and the chair of the hospital district waived the need for written informed patient consent due to the retrospective nature of the study. All clinical investigations were conducted according to the relevant guidelines and the principles expressed in the Declaration of Helsinki.

### Study population and screening protocol

Finland was the first country in the world to undertake nationwide organized screening for breast cancer in 1987^36^. Municipalities are obliged by law to organize screening services or purchase them from a private provider. A personal letter is sent every 2 years to all women in the target age range (50–69 years), inviting them to participate in screening. Mammography and recall examinations are free of charge for all participants. Generally screening logistics are uniform across the municipalities, mammograms are assessed in a double blinded reading manner with subsequent consensus reading by two independent readers and recall assessments are performed at the same unit as performing the screening examination, however there are differences in imaging equipment that is used by different screening providers.

Patients were referred to our hospital for consultation and treatment from four screening units at different time points, including from two district hospitals and multiple primary healthcare centers representing a catchment area of approximately 250,000 people. In general, basic breast examinations were performed as part of recall assessment upon referral, including a two-view mammogram and bilateral whole-breast and axillary ultrasound examinations. The images of all patients are stored in the local picture archiving and communication system (PACS).

The study population consisted of 942 female patients aged 50–69 years and living in the North-Savo region, with breast cancer treated and followed-up at Kuopio University Hospital (Kuopio, Finland) between January 2010 and December 2016. Patients with lesions invisible on mammography (60 women) were excluded from the study, so a total of 882 women with breast cancer were included.

### Assessment of radiological features

All patients’ medical records are stored in the local digital archive, from which the data were retrieved for the study. The data retrieved from the medical records included the patient’s age, histopathological features and diagnosis, surgical and oncological treatments, recurrence, and date and cause of death. The radiological features on mammograms stored in the PACS were assessed by one of two specialist breast radiologists with over 15 years of experience, in a blind, independent manner. The radiological features assessed included the lesion’s diameter, breast density according to the 4th and 5th editions of the Breast Imaging-Reporting and Data System (BI-RADS)^37,38^, local breast density around the tumor (visual estimation of fibroglandular tissue around the tumor that might obscure tumor and margins) and lesion type. The mammographic tumor characteristics were evaluated according to the 5th edition of BI-RADS. For the classification of true, minimal-signs, missed, and occult cancers, one of the two radiologists initially assessed the images, and later all missed and most minimal-signs cancers were re-evaluated in consensus by the same radiologists. Cancers that were not visible on previous screening mammograms were classified as true cancers; cancers that showed nonspecific minor abnormalities on previous screening mammograms that did not lead to a diagnosis of breast cancer were classified as minimal-signs cancers; cancers that were visible on a previous screening mammogram but were overlooked were classified as missed cancers; and cancers that were not mammographically visible at diagnosis were classified as occult cancers. Cancers were deemed “missed” only if both experts agreed on the classification. Both screening-detected and interval cancers were divided into the four categories.

Three perpendicular tumor diameters were measured, and the tumor volume was estimated using the formula for oblate spheroids: V = 4/3π × a/2 × b/2 × c/2, where a, b, and c denote the longest diameter of the lesion, the maximum perpendicular diameter in the same plane, and the longest vertical diameter in the orthogonal plane, respectively^39^. The tumor growth rate was estimated for missed cancers by comparing two mammograms taken at different times. The specific growth rate (SGR, %/day) was calculated using the following equation: SGR = ln(V2/V1)/(t2 − t1), and the doubling time (DT) was calculated as: DT = (t2 − t1)ln2/ln(V2/V1), where V1 and V2 are the tumor volumes at the time of the first mammogram (t1) and the second mammogram (t2), respectively^40^.

All statistical analyses were performed using SPSS version 27 for Windows (IBM Corporation, Armonk, NY, USA). The local breast density around the tumors was assessed visually and recorded on a continuous percentage scale of 0–100%. A χ^2^ test was used to compare the radiological and pathological variables between the breast cancer subgroups. An unpaired *t* test was used to evaluate the differences in the growth rates of missed and minimal-signs cancers. The Kaplan–Meier estimator was used to examine the survival of patients with screening-detected or interval cancers. Survival analyses were repeated also after the inclusion of occult cancers. *p* values of < 0.05 were considered statistically significant.

### Ethics approval

In accordance with the Finnish national regulations (Medical Research Act, 488/1999), ethics committee approval is not mandatory in retrospective registry studies and the chair of the hospital district waived the need for written informed patient consent due to the retrospective nature of the study (permission number 74/2020 and registrar permission number 67/2020). All clinical investigations were conducted according to the relevant guidelines and the principles expressed in the Declaration of Helsinki.

## Results

A total of 882 breast cancer patients (mean age 60.4 ± 5.5 years) were included in the study, in whom 581 cancers were screening-detected and 301 (34.1%) were interval cancers. Most breast cancers (96.4%) occurred in fatty low-density breasts with area percentage ≤ 50% when density was assessed according to the 4th edition of BI-RADS, whereas 62.6% of breast cancers occurred in fatty breasts and breasts with scattered areas of fibroglandular density (categories A and B) when density was assessed according to the 5th edition of BI-RADS. The most frequent lesion types were masses (73.9%) and calcification (13.4%), whereas distortion (1.8%) and asymmetries (1.7%) were least common.

A comparison of the radiological features of screening-detected and interval cancers is shown in Table 1. The screening-detected cancers were smaller than the interval cancers on mammograms. The largest screening-detected cancers were observed in the true group (mean 19.5 mm), whereas the largest interval cancers were observed in the missed group (mean 28.6 mm). Interval cancers were observed more frequently than screening-detected cancers in denser breasts, especially when density was assessed according to the 5th edition of BI-RADS. Masses (74.2%) and calcifications (15.3%) were the most common lesion types observed in screening-detected cancers, whereas masses (73.4%) and masses with calcification (11.2%) were the most common lesion types among interval cancers. The local breast density around the tumors was not significantly different between groups (*p* = 0.306).

**Table 1:** Comparison of radiological features of screening-detected and interval cancers.

	Screening-detected cancer								Interval cancer							
	Missed		Minimal-signs		True		No previous MGR available		Missed		Minimal-signs		True		No previous MGR available	
	n = 99		n = 124		n = 245		n = 113		n = 28		n = 46		n = 98		n = 129	
	N	(%)	N	(%)	N	(%)	N	(%)	N	(%)	N	(%)	N	(%)	N	(%)
Mammographic diameter (mm)																
Mean ± SD	19.1 ± 11.3		17.3 ± 10.0		19.5 ± 15.9		18.6 ± 11.6		28.6 ± 20.4		26.0 ± 17.1		25.4 ± 16.8		25.5 ± 18.4	
Median (IQR)	16 (12, 23)		15 (12, 20)		14 (11, 22)		15 (12, 23)		22 (18, 31)		19 (15, 34)		20 (16, 29)		21 (15, 30)	
Breast density (BI-RADS 4th edition)																
1	80	80.8	104	83.9	196	80.0	80	70.8	24	85.7	32	69.6	72	73.5	93	72.1
2	17	17.2	15	12.1	42	17.1	29	25.7	4	14.3	10	21.7	24	24.5	28	21.7
3	2	2.0	5	4.0	7	2.9	3	2.7	0	0.0	4	8.7	1	1.0	6	4.7
4	0	0.0	0	0.0	0	0.0	1	0.9	0	0.0	0	0.0	1	1.0	2	1.6
Total	99		124		245		113		28		46		98		129	
Breast density (BI-RADS 5th edition)																
A	17	17.2	26	21.0	37	15.1	15	13.3	4	14.3	3	6.5	5	5.1	22	17.1
B	50	50.5	56	45.2	128	52.2	58	51.3	13	46.4	14	30.4	45	45.9	59	45.7
C	31	31.3	41	33.1	79	32.2	38	33.6	11	39.3	27	58.7	47	48.0	46	35.7
D	1	1.0	1	0.8	1	0.4	2	1.8	0	0.0	2	4.3	1	1.0	2	1.6
Total	99		124		245		113		28		46		98		129	
Local breast density around tumor (%)																
Mean ± SD	39.1 ± 34.4		43.1 ± 33.8		44.0 ± 36.5		41.1 ± 34.7		43.0 ± 28.4		59.4 ± 34.8		56.4 ± 35.5		51.3 ± 38.7	
Median (IQR)	30 (10, 60)		40 (10, 70)		40 (10, 70)		30 (10, 65)		45 (21, 60)		60 (24, 100)		60 (25, 100)		50 (11, 100)	
Lesion type																
Mass	65	65.7	105	84.7	178	72.7	83	73.5	18	64.3	35	76.1	70	71.4	98	76.0
Mass with calcification	9	9.1	7	5.6	17	6.9	14	12.4	7	25.0	6	13.0	9	9.2	12	9.3
Calcification	25	25.3	9	7.3	40	16.3	15	13.3	3	10.7	4	8.7	7	7.1	15	11.6
Asymmetry	0	0.0	1	0.8	4	1.6	0	0.0	0	0.0	1	2.2	8	8.2	1	0.8
Distortion	0	0.0	2	1.6	6	2.4	1	0.9	0	0.0	0	0.0	4	4.1	3	2.3
Total	99		124		245		113		28		46		98		129	
Mass																
Shape																
Oval	28	37.8	45	40.2	61	31.3	38	39.2	8	32.0	14	34.1	25	31.6	42	38.2
Round	16	21.6	23	20.5	68	34.9	27	27.8	6	24.0	11	26.8	25	31.6	35	31.8
Irregular	30	40.5	44	39.3	66	33.8	32	33.0	11	44.0	16	39.0	29	36.7	33	30.0
Total	74		112		195		97		25		41		79		110	
Margins																
Circumscribed	1	1.4	5	4.5	7	3.6	0	0.0	1	4.0	3	7.3	4	5.1	5	4.5
Obscured	4	5.4	9	8.0	11	5.6	7	7.2	1	4.0	8	19.5	14	17.7	6	5.5
Microlobulated	13	17.6	18	16.1	63	32.3	29	29.9	5	20.0	4	9.8	19	24.1	40	36.4
Indistinct	11	14.9	25	22.3	52	26.7	24	24.7	3	12.0	12	29.3	24	30.4	27	24.5
Spiculated	45	60.8	55	49.1	62	31.8	37	38.1	15	60.0	14	34.1	18	22.8	32	29.1
Total	74		112		195		97		25		41		79		110	
Calcifications																
Morphology																
Punctate	0	0.0	0	0.0	0	0.0	0	0.0	0	0.0	0	0.0	0	0.0	1	3.7
Amorphous	6	17.6	2	12.5	3	5.3	1	3.4	0	0.0	1	10.0	1	6.3	2	7.4
Coarse heterogeneous	1	2.9	0	0.0	3	5.3	6	20.7	2	20.0	0	0.0	1	6.3	1	3.7
Fine pleomorphic	15	44.1	13	81.3	38	66.7	16	55.2	7	70.0	7	70.0	10	62.5	12	44.4
Fine linear or branching	12	35.3	1	6.3	13	22.8	6	20.7	1	10.0	2	20.0	4	25.0	11	40.7
Total	34		16		57		29		10		10		16		27	
Distribution																
Diffuse	2	5.9	0	0.0	3	5.3	0	0.0	1	10.0	1	10.0	0	0.0	0	0.0
Regional	10	29.4	2	12.5	10	17.5	3	10.3	4	40.0	0	0.0	3	18.8	9	33.3
Grouped	12	35.3	12	75.0	36	63.2	21	72.4	5	50.0	9	90.0	9	56.3	18	66.7
Linear	4	11.8	1	6.3	2	3.5	0	0.0	0	0.0	0	0.0	0	0.0	0	0.0
Segmental	6	17.6	1	6.3	6	10.5	5	17.2	0	0.0	0	0.0	4	25.0	0	0.0
Total	34		16		57		29		10		10		16		27	

MGR, Mammography; mm, Millimeter; SD, Standard deviation; IQR; Interquartile range; BI-RADS, Breast Imaging-Reporting and Data System.

The histopathologically determined tumor sizes differed significantly among the groups, as seen in Table 2. Missed cancers were smallest (mean 17.2 mm), whereas true cancers were largest (mean 19.7 mm).

**Table 2:** Overall histopathological characteristics.

	Missed		Minimal-signs		True		No previous MGR available		Total	*p* value
	N	(%)	N	(%)	N	(%)	N	(%)		
Tumor size (mm)										
Mean ± SD	17.2 ± 12.1		18.4 ± 14.0		19.7 ± 15.5		21.0 ± 16.5		0.042	
Median (IQR)	15 (10, 20)		15 (10, 22)		15 (10, 25)		16 (12, 25)			
Histopathological type										
Ductal carcinoma	85	66.9	125	73.5	240	70.0	177	73.1	627	0.311
Lobular carcinoma	15	11.8	24	14.1	47	13.7	23	9.5	109	
DCIS	18	14.2	9	5.3	34	9.9	13	5.4	74	
Other cancer	9	7.1	12	7.1	22	6.4	29	12.0	72	
Total	127		170		343		242		882	
T										
Tis	18	14.2	9	5.3	34	9.9	14	5.8	75	0.062
1	83	65.4	113	66.5	207	60.3	145	59.9	548	
2	22	17.3	41	24.1	86	25.1	65	26.9	214	
3	3	2.4	3	1.8	12	3.5	7	2.9	25	
4	1	0.8	4	2.4	3	0.9	11	4.5	19	
Tx	0	0.0	0	0.0	1	0.3	0	0.0	1	
Total	127		170		343		242		882	
N										
0	91	71.7	116	68.2	243	70.8	158	65.3	608	0.464
1	24	18.9	38	22.4	63	18.4	55	22.7	180	
2	4	3.1	11	6.5	24	7.0	13	5.4	52	
3	8	6.3	4	2.4	13	3.8	16	6.6	41	
Nx	0	0.0	1	0.6	0	0.0	0	0.0	1	
Total	127		170		343		242		882	
M										
0	122	96.1	154	90.6	304	88.7	213	88.4	793	0.156
1	5	3.9	15	8.2	39	11.3	28	11.2	87	
Mx	0	0.0	1	1.2	0	0.0	1	0.4	2	
Total	127		170		343		242		882	
Grade										
1	36	28.3	45	26.5	81	23.6	45	18.6	207	0.001
2	62	48.8	97	57.1	156	45.5	113	46.7	428	
3	27	21.3	27	15.9	98	28.6	83	34.3	235	
Data missing	2	1.6	1	0.6	8	2.3	1	0.4	12	
Total	127		170		343		242		882	
ER										
Positive	106	83.5	154	90.6	274	79.9	198	81.8	732	0.003
Negative	3	2.4	7	4.1	33	9.6	27	11.2	70	
DCIS	18	14.2	9	5.3	34	9.9	13	5.4	74	
Data missing	0	0.0	0	0.0	2	0.6	4	1.7	6	
Total	127		170		343		242		882	
PR										
Positive	102	80.3	151	88.8	274	79.9	198	81.8	725	0.147
Negative	7	5.5	10	5.9	33	9.6	27	11.2	77	
DCIS	18	14.2	9	5.3	34	9.9	13	5.4	74	
Data missing	0	0.0	0	0.0	2	0.6	4	1.7	6	
Total	127		170		343		242		882	
HER2										
Positive	15	11.8	14	8.2	56	16.3	43	17.8	128	0.022
Negative	94	74.0	147	86.5	251	73.2	182	75.2	674	
DCIS	18	14.2	9	5.3	34	9.9	13	5.4	74	
Data missing	0	0.0	0	0.0	2	0.6	4	1.7	6	
Total	127		170		343		242		882	
Ki­67										
Lower (0–19%)	57	44.9	83	48.8	140	40.8	105	43.4	385	0.507
Higher (20–100%)	52	40.9	78	45.9	167	48.7	116	47.9	413	
DCIS	18	14.2	9	5.3	34	9.9	13	5.4	74	
Data missing	0	0.0	0	0.0	2	0.6	8	3.3	10	
Total	127		170		343		242		882	

MGR, Mammography; mm, Millimeter; SD, Standard deviation; IQR, Interquartile range; DCIS, Ductal carcinoma in situ; ER, Estrogen receptor; PR, Progesterone receptor; HER2, Human epidermal growth factor receptor 2.

Histopathological assessment showed that screening-detected cancers were smaller than interval cancers (Table 3). Cancers were most frequently grade 2 in every category for both screening-detected and interval cancers, but there were more grade 3 cancers among the interval cancers (38.2%) than among the screening-detected cancers (20.7%). Interval cancers were more often HER2-positive (19.6% vs. 12.0%) with higher Ki-67 indices than screening-detected cancers (60.1% vs. 40.1%), and patients with interval cancers were more frequently node-positive (45.5% vs. 23.4%) and had more distant metastases than patients with screening-detected cancers (18.6% vs. 5.3%).

**Table 3:** Comparison of histopathological characteristics of screening-detected and interval cancers.

	Screening-detected cancer								Interval cancer							
	Missed		Minimal-signs		True		No previous MGR available		Missed		Minimal-signs		True		No previous MGR available	
	n = 99		n = 124		n = 245		n = 113		n = 28		n = 46		n = 98		n = 129	
	N	(%)	N	(%)	N	(%)	N	(%)	N	(%)	N	(%)	N	(%)	N	(%)
Tumor size (mm)																
Mean ± SD	15.4 ± 10.3		16.1 ± 10.4		17.7 ± 14.6		16.5 ± 10.3		23.1 ± 15.9		24.9 ± 19.6		24.5 ± 16.6		24.9 ± 19.6	
Median (IQR)	15 (10, 20)		13 (10, 20)		13 (10, 21)		15 (10, 20)		20 (12, 29)		17 (13, 36)		20 (13, 30)		20 (14, 30)	
Histopathological type																
Ductal carcinoma	64	64.6	95	76.6	179	73.1	86	76.1	21	75.0	30	65.2	61	62.2	91	70.5
Lobular carcinoma	11	11.1	15	12.1	27	11.0	11	9.7	4	14.3	9	19.6	20	20.4	12	9.3
DCIS	17	17.2	8	6.5	28	11.4	6	5.3	1	3.6	1	2.2	6	6.1	7	5.4
Other cancer	7	7.1	6	4.8	11	4.5	10	8.8	2	7.1	6	13.0	11	11.2	19	14.7
Total	99		124		245		113		28		46		98		129	
T																
Tis	17	17.2	8	6.5	28	11.4	7	6.2	1	3.6	1	2.2	6	6.1	7	5.4
1	69	69.7	89	71.8	163	66.5	79	69.9	14	50.0	24	52.2	44	44.9	66	51.2
2	11	11.1	25	20.2	46	18.8	24	21.2	11	39.3	16	34.8	40	40.8	41	31.8
3	1	1.0	1	0.8	8	3.3	1	0.9	2	7.1	2	4.3	4	4.1	6	4.7
4	1	1.0	1	0.8	0	0.0	2	1.8	0	0.0	3	6.5	3	3.1	9	7.0
Tx	0	0.0	0	0.0	0	0.0	0	0.0	0	0.0	0	0.0	1	1.0	0	0.0
Total	99		124		245		113		28		46		98		129	
N																
0	78	78.8	92	74.2	186	75.9	88	77.9	13	46.4	24	52.2	57	58.2	70	54.3
1	16	16.2	27	21.8	44	18.0	20	17.7	8	28.6	11	23.9	19	19.4	35	27.1
2	2	2.0	4	3.2	14	5.7	1	0.9	2	7.1	7	15.2	10	10.2	12	9.3
3	3	3.0	0	0.0	1	0.4	4	3.5	5	17.9	4	8.7	12	12.2	12	9.3
Nx	0	0.0	1	0.8	0	0.0	0	0.0	0	0.0	0	0.0	0	0.0	0	0.0
Total	99		124		245		113		28		46		98		129	
M																
0	98	99.0	116	93.5	230	93.9	105	92.9	24	85.7	38	82.6	74	75.5	108	83.7
1	1	1.0	7	5.6	15	6.1	8	7.1	4	14.3	8	17.4	24	24.5	20	15.5
Data missing	0	0.0	1	0.8	0	0.0	0	0.0	0	0.0	0	0.0	0	0.0	1	0.8
Total	99		124		245		113		28		46		98		129	
Grade																
1	31	31.3	39	31.5	66	26.9	28	24.8	5	17.9	6	13.0	15	15.3	17	13.2
2	45	45.5	70	56.5	112	45.7	63	55.8	17	60.7	27	58.7	44	44.9	50	38.8
3	21	21.2	14	11.3	63	25.7	22	19.5	6	21.4	13	28.3	35	35.7	61	47.3
Data missing	2	2.0	1	0.8	4	1.6	0	0.0	0	0.0	0	0.0	4	4.1	1	0.8
Total	99		124		245		113		28		46		98		129	
ER																
Positive	81	81.8	114	91.9	198	80.8	98	86.7	25	89.3	40	87.0	76	77.6	100	77.5
Negative	1	1.0	2	1.6	17	6.9	6	5.3	2	7.1	5	10.9	16	16.3	22	17.1
DCIS	17	17.2	8	6.5	28	11.4	6	5.3	1	3.6	1	2.2	6	6.1	7	5.4
Data missing	0	0.0	0	0.0	2	0.8	3	2.7	0	0.0	0	0.0	0	0.0	0	0.0
Total	99		124		245		113		28		46		98		129	
PR																
Positive	76	76.8	110	88.7	193	78.8	98	86.7	26	92.9	41	89.1	81	82.7	101	78.3
Negative	6	6.1	6	4.8	22	9.0	6	5.3	1	3.6	4	8.7	11	11.2	21	16.3
DCIS	17	17.2	8	6.5	28	11.4	6	5.3	1	3.6	1	2.2	6	6.1	7	5.4
Data missing	0	0.0	0	0.0	2	0.8	3	2.7	0	0.0	0	0.0	0	0.0	0	0.0
Total	99		124		245		113		28		46		98		129	
HER2																
Positive	8	8.1	9	7.3	37	15.1	16	14.2	7	25.0	5	10.9	19	19.4	28	21.7
Negative	74	74.7	107	86.3	178	72.7	88	77.9	20	71.4	40	87.0	73	74.5	94	72.9
DCIS	17	17.2	8	6.5	28	11.4	6	5.3	1	3.6	1	2.2	6	6.1	7	5.4
Data missing	0	0.0	0	0.0	2	0.8	3	2.7	0	0.0	0	0.0	0	0.0	0	0.0
Total	99		124		245		113		28		46		98		129	
Ki-67																
Lower (0–19%)	44	44.4	66	53.2	113	46.1	60	53.1	13	46.4	17	37.0	27	27.6	45	34.9
Higher (20–100%)	38	38.4	50	40.3	102	41.6	43	38.1	14	50.0	28	60.9	65	66.3	74	57.4
DCIS	17	17.2	8	6.5	28	11.4	6	5.3	1	3.6	1	2.2	6	6.1	7	5.4
Data missing	0	0.0	0	0.0	2	0.8	4	3.5	0	0.0	0	0.0	0	0.0	3	2.3
Total	99		124		245		113		28		46		98		129	

MGR, Mammography; mm, Millimeter; SD, Standard deviation; IQR, Interquartile range; DCIS, Ductal carcinoma in situ; ER, Estrogen receptor; PR, Progesterone receptor; HER2, Human epidermal growth factor receptor 2.

Patients with true interval cancer included fewer cases of positive nodal status than patients with other types of interval cancer. However, patients with true interval cancer had the highest proportion of high-Ki-67 cancers and distant metastasis of all groups of patients with either interval or screening-detected cancers.

The growth rates in missed and minimal-signs cancers are shown in Table 4. Overall, there was no statistically significant difference in the growth rates of missed and minimal-signs cancers. Missed and minimal-signs interval cancers had faster specific growth rates than screening-detected cancers. The doubling times were shorter in missed and minimal-signs interval cancers than in missed and minimal-signs screening-detected cancers.

**Table 4:** Growth rates in missed and minimal-signs screening-detected and interval cancers.

	Screening-detected cancer		Interval cancer	
	Missed	Minimal-signs	Missed	Minimal-signs
N	99	124	28	46
Time between previous mammogram and diagnosis (days)				
Mean ± SD	755.0 ± 195.1	758.9 ± 187.1	521.1 ± 221.3	482.9 ± 223.1
Specific growth rate (%/day)				
Mean ± SD	0.21 ± 0.11	0.27 ± 0.13	0.47 ± 0.77	0.48 ± 0.38
Doubling time (days)				
Mean ± SD	400.8 ± 392.4	353.5 ± 287.0	224.8 ± 607.4	155.5 ± 764.0

The screening providers varied between municipalities and over time. There was an increasing trend in the numbers of missed cancers in the beginning of the 2010s: 9 missed cancers in 2010, 10 in 2011, 19 in 2012, and peaking in 2013 with 43 missed cancers. Nearly all missed cancers were due to human error (95.3%), whereas only 1.6% were attributable to technical issues and 3.1% to both human error and technical issues.

The treatment distribution among the screening-detected and interval cancers is shown in Table 5. Nearly all screening-detected cancers were surgically operable and underwent surgical treatment (99.3%), whereas interval cancers were not treated surgically to the same degree (95.7%). Patients with interval cancers received postoperative chemotherapy noticeably more often and postoperative hormonal treatment slightly more often than those with screening-detected cancers. Screening-detected cancers were more frequently treated with postoperative radiotherapy than were interval cancers.

**Table 5:** Comparison of treatment distributions in screening-detected and interval cancers.

	Screening-detected cancer		Interval cancer		*p* value
	N	(%)	N	(%)	
Surgery	577	99.3	288	95.7	< 0.001
Chemotherapy	266	45.8	189	62.8	< 0.001
Radiotherapy	502	86.4	237	78.7	< 0.001
Hormonal treatment	423	72.8	225	74.8	0.589

**Figure 1:**
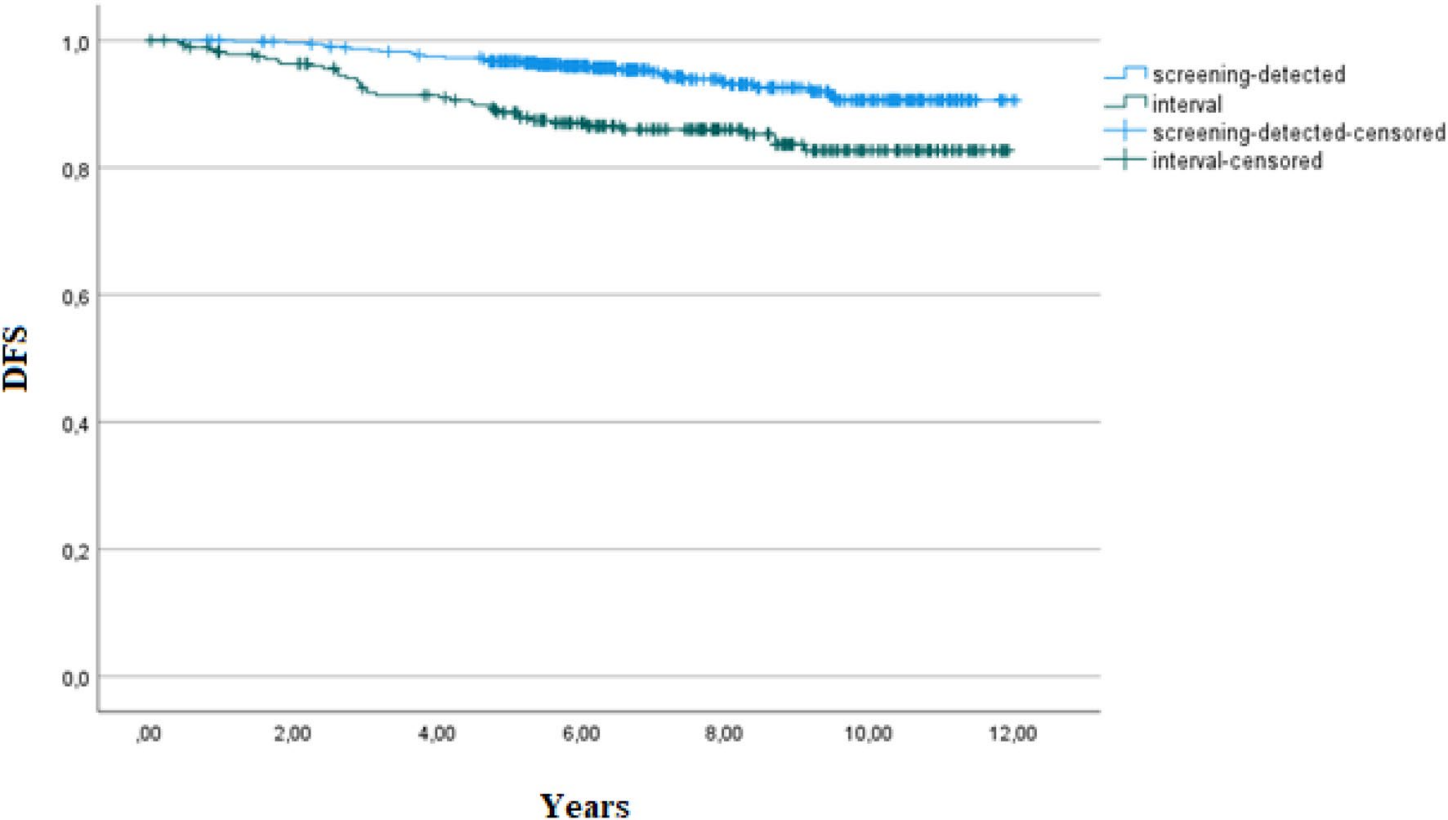
Kaplan–Meier estimates of disease-free survival in screening-detected and interval cancers.

The mean follow-up time for this study population was 8.2 years. Patients with ductal carcinoma in situ (DCIS) or a history of previous breast cancer (n = 10) were not included in the survival analysis. Patients with no previous mammogram available were included in the survival analyses. We observed a statistically significant difference in disease-free survival (DFS) between patients with screening-detected and those with interval cancers, as shown in Table 6 and Fig. 1. DFS was significantly worse in patients with interval cancer than in those with screening-detected cancers. Within subgroups of cancers (true, minimal-signs, or missed) there was no statistically significant difference in DFS between screening-detected or interval cancers.

**Table 6:** The absolute amount and proportions of endpoints in survival analyses for screening-detected and interval cancers.

	Screening-detected cancers N (%)	Interval cancers N (%)	*p* value
Disease-free survival	32 (5.5)	40 (13.3)	< 0.001
Overall survival	39 (6.7)	57 (18.9)	< 0.001
Disease-specific survival	17 (2.9)	39 (13.0)	< 0.001

**Figure 2:**
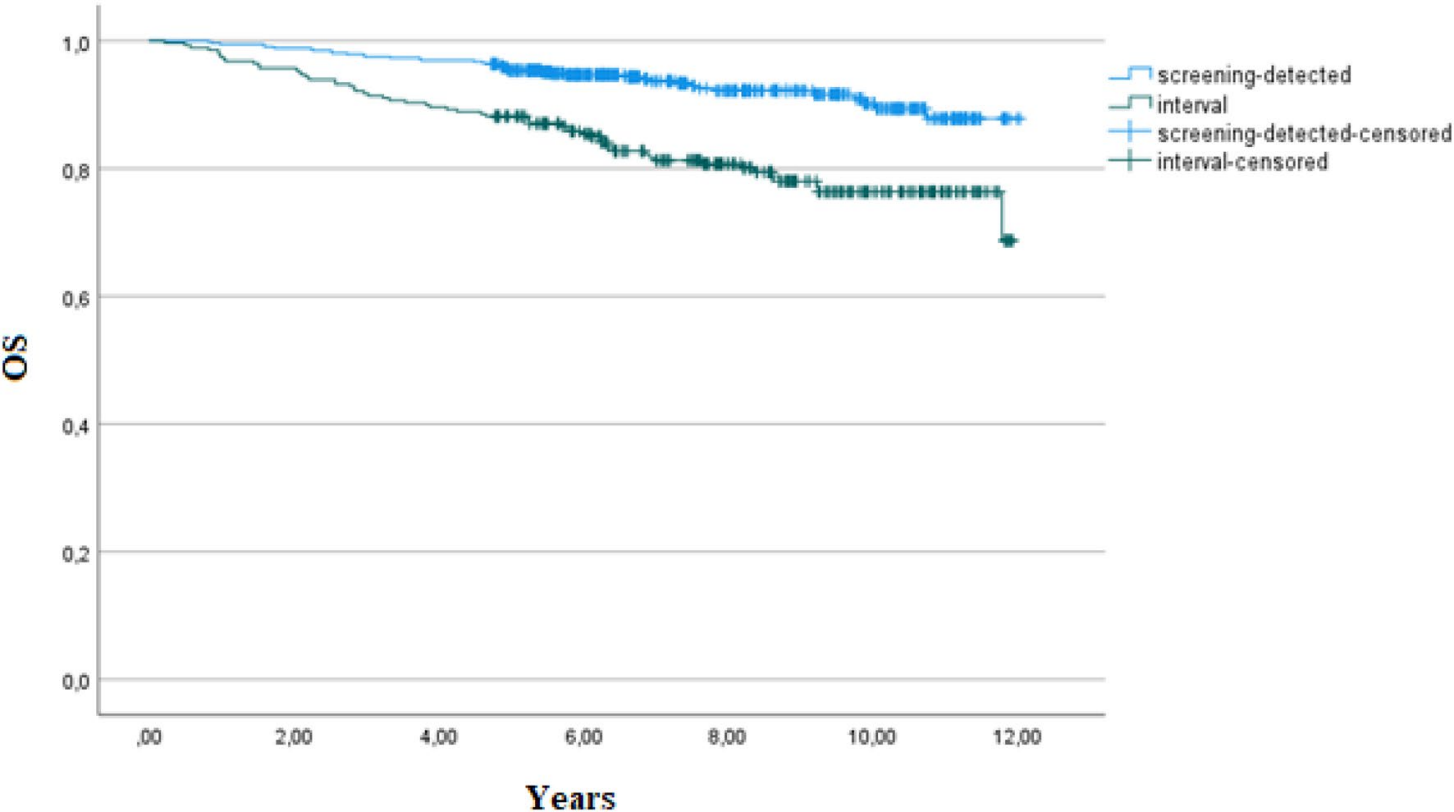
Kaplan–Meier estimates of overall survival in screening-detected and interval cancers.

**Figure 3:**
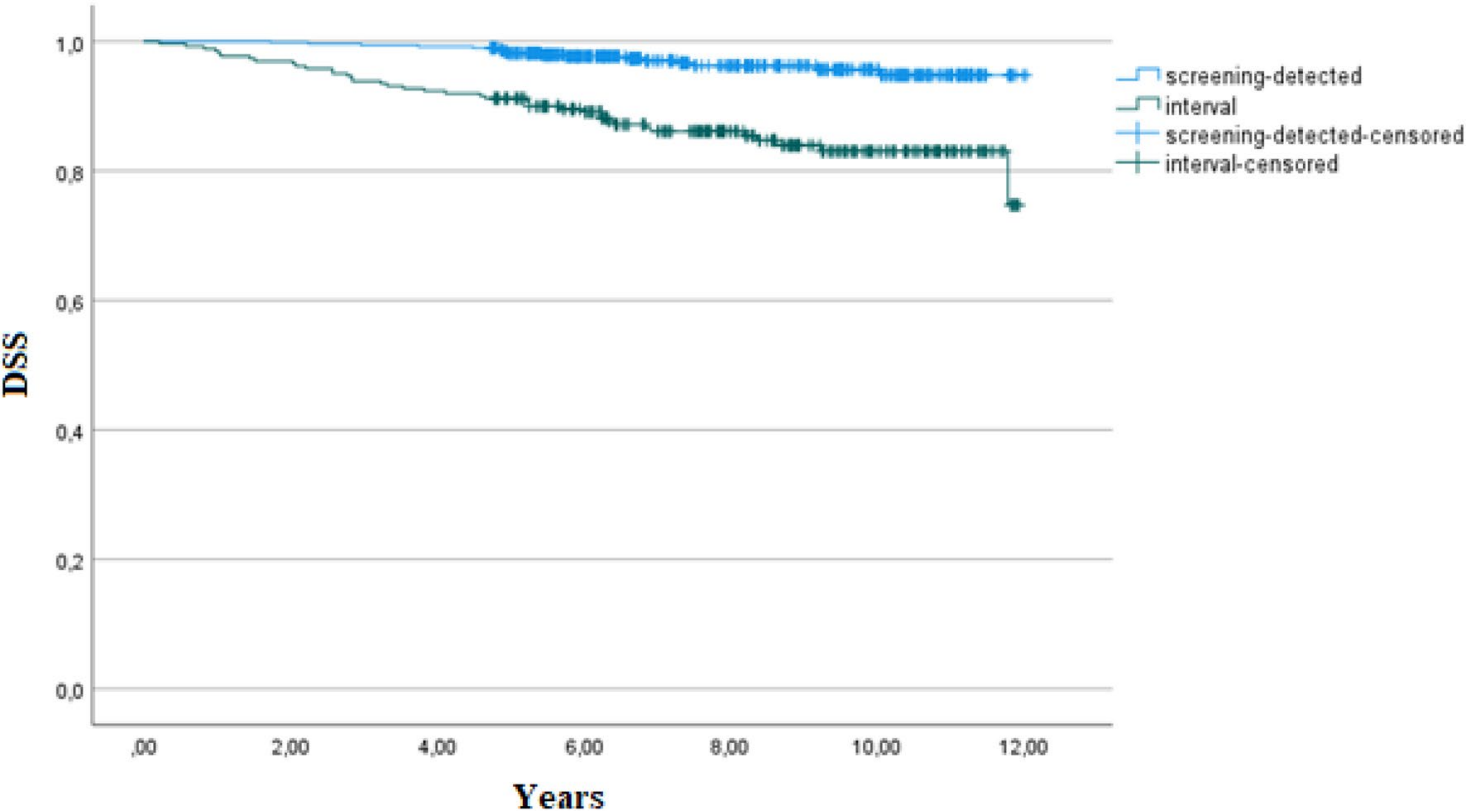
Kaplan–Meier estimates of disease-specific survival in screening-detected and interval cancers.

We also observed a statistically significant difference in overall survival (OS) and in disease-specific survival (DSS) between patients with screening-detected and interval cancers, as shown in Table 6 and Figs. 2 and 3. OS and DSS was significantly worse in patients with interval cancer. Within subgroups of cancers (true, minimal-signs, or missed) there was no statistically significant difference in OS or in DSS between screening-detected or interval cancers.

Statistical significance (*p* = 0.001) of the difference between interval cancers and screening-detected cancers was preserved also after the inclusion of all occult cancers in the survival analyses (DFS, OS and DSS).

## Discussion

In the present study we assessed the radiological and histopathological features and growth rates of screening-detected and interval breast cancers and the subsequent survival rates in an eEastern Finnish population. Survival was worse, histopathological features were more aggressive and growth rates were faster in interval cancers when compared to screening-detected cancers. There was a marked difference in treatment distributions between interval and screening-detected cancers. The proportion of interval cancers in the studied population was within the reported range when compared to other reports, however there were distinct differences in the amount of missed cancers between screening providers. The radiological features of the studied population differed from previous reports.

In this study, with a relatively long follow-up period, we observed a statistically significant difference in DFS, OS and DSS between patients with screening-detected and interval cancers. Those with interval cancer had significantly worse DFS, OS and DSS, indicating that interval cancer is more aggressive. Therefore, our results confirm the worse outcome of interval cancer. Previous reports of survival after interval cancer have shown differing results. Studies have not uniformly been able to demonstrate worse survival in interval cancers compared to screening-detected cancers. Previous reports of survival after interval cancer are usually based on old data with widely varying follow-up time, so direct comparison is challenging. Most studies have shown that interval cancer has worse OS than screening-detected cancer^18,21,24,27,29–32,34^, the other studies found no significant difference^19,22,26,35^. Additionally, only few reports have previously investigated the DFS associated with interval cancers and reported worse outcome compared to screening-detected cancers^30,31^. The endpoint events observed in this study, 39 (6.7%) deceased and 32 (5.5%) recurrences among screening-detected and 57 (18.9%) deceased and 40 (13.3%) recurrences among interval cancer patients, are similar to other recent reports where the amount of deceased range from 8 to 188 (4.2–18.4%) among screening-detected and from 8 to 191 (10.0–27.6%) among interval cancer patients and recurrences have been observed to range from 8 to 16 (3.7–12.4%) among screening-detected and from 8 to 11 (10.4–23.5%) among interval cancer patients^3,31,32,34,35^.

Previous reports have shown no difference in survival among the subgroups of interval cancer, except for the study by Fong et al.^3,20,21,23,25,32,33^. In that study, the researchers examined the overall 10-year survival of patients with interval cancer and compared the OS of screening-detected and interval cancer patients^32^. Survival was significantly worse in patients with interval cancer than in those with screening-detected cancer, and missed and occult interval cancer entailed significantly worse long-term survival than true interval cancer^32^. Tsuruda et al. were the first group to study OS within subgroups of screening-detected cancer, and they detected no differences during the 5-year follow-up period^3^. Our results are consistent with the majority of reports insofar as we observed no significant difference in DFS or OS between the subgroups of interval cancer, even over a long follow-up period, probably because the subgroup populations were small so the analysis lacked statistical power.

Nearly all the screening-detected cancers were treated surgically, whereas the interval cancers were not treated surgically to the same degree, which may be explained by the fact that interval cancers are more distantly spread at baseline, which excludes surgical treatment at the outset. Interval cancers also received more postoperative treatments in terms of chemotherapy and hormonal therapy than the screening-detected cancers. However, survival was still worse, although such treatments have been claimed to reduce mortality rates and improve survival in patients with interval cancer^27,28,41^.

The number of interval cancers in a population depends on the review method used and has been reported in different settings to range from 17.7–35.9% of breast cancers^3,10,11,21,23,32,33^. Our interval cancer proportion of 34.1% was within the reported range. It is likely that many of these missed cancers could have been detected earlier, emphasizing the importance of a high-quality screening program. We noted that nearly all the missed cancers were attributable to human error, so additional training and a better knowledge of the radiological features of breast cancers in a population should reduce the numbers of missed cancers. Research data are required to address the problem of program quality and the regional inequalities in screening programs in the context of the social and health-care reform that was recently launched in Finland. Our results stress the need for the better auditing and control of the regional screening program.

Interval breast cancers differ in many respects from screening-detected cancers. Interval cancers are more often triple negative and human epidermal growth factor receptor 2 (HER2)-positive, with higher Ki-67, whereas phenotypes luminal A and B are more common in screening-detected cancers. Interval cancers are larger, have a higher histological grade and a greater frequency of node metastases, and are of a more advanced stage than screening-detected cancers^5,17,42^. The radiographic features that are more common in screening-detected cancers than in interval cancers include masses, distortions, and calcification, whereas asymmetries, masses with calcification, and distortions with calcification are more common in interval cancers. Masses, calcification, and asymmetries are more common in true interval cancers than in missed interval cancers, whereas distortions, masses with calcification, and distortions with calcification are more common in missed interval cancers than in true interval cancers^42^. Interval breast cancers in low-density breasts (breast density < 20–25%) have a particularly aggressive phenotype and a worse prognosis than screening-detected breast cancers in low-density breasts, whereas interval cancers in denser breasts (breast density ≥ 50%) are phenotypically more similar to screening-detected cancers^4,17^.

In the present study, the population radiological features differed slightly from those in previous reports^5,42^. Calcification was observed more often in screening-detected cancers than in interval cancers, whereas masses with calcification and asymmetries were observed more often in interval cancers. In our study population, the typical screening-detected cancers were masses with irregular or oval shapes and spiculated or microlobulated margins. The typical interval cancers were masses with irregular shapes and spiculated or indistinct margins.

Overall, we observed statistically significant differences in the histopathological tumor sizes between the groups. Missed cancers were interestingly smaller than cancers in other subgroups in both screening-detected and interval cancers whereas previous studies have reported that missed interval cancers have been larger than true interval cancers^5^. Clear distinctions in growth rates and doubling times were seen between the missed/minimal-signs screening-detected and interval cancers. Interval breast cancers have been associated with faster growth rates in previous reports^21^. The missed and minimal-signs interval cancers had faster specific growth rates and lower doubling times, which were sufficient to cause symptoms that allowed the cancer to be detected before the next screening. Interval cancers were also noticeably more aggressive in terms of nodal and distant metastases as has been previously reported^5^. A positive nodal status was observed in up to 53.6% of missed interval cancers, which had the highest level of nodal spreading among all interval cancer subgroups, even though missed cancers were the smallest interval cancers. Distant metastases were noted more often in patients with interval cancers than in those with screening-detected cancers. Up to 24.5% of true interval cancers had distant metastases, whereas the greatest amount of distant metastases in screening-detected cancers was observed in the true category (6.1%) and only 1.0% of missed screening-detected cancers involved distant metastases. Interestingly, true cancers had the highest numbers of distant metastases in both the screening-detected and interval cancers, indicating that true cancers were most aggressive, although a positive nodal status was seen more often in the other subgroups. True cancers more frequently displayed higher Ki-67 levels than the other subgroups and, overall, interval cancers had higher Ki-67 levels than screening-detected cancers. Interval cancers were more often HER2-positive than screening-detected cancers, and although the vast majority of cancers were ER- and PR-positive, true interval cancers had the lowest rates of ER and PR positivity of all the interval cancers. Histopathological features of interval cancers in the studied population are similar to previous reports^5^.

There was no marked difference in the distribution of breast density between screening-detected and interval cancers when density was assessed according to the 4th edition of BI-RADS. However, a marked difference was observed when assessments were made according to the 5th edition of BI-RADS, when interval cancers were categorized as denser than screening-detected cancers. The density distribution of our population also differed from those previously reported because the majority of breast cancers occurred in low-density breasts^43^.

This study had several limitations. Screening-detected cancers benefit from the length time bias, and longer follow-up periods are required to reduce the influence of lead time bias on survival^44^. Moreover, this retrospective review was not totally blinded because the observers were aware that all patients reviewed had been diagnosed with cancer because the protocol did not include screening images without cancer. Nevertheless, the aim of the review was the retrospective evaluation of the numbers and outcomes of cancers that were deemed diagnosable (true or with minimal signs) by expert breast radiologists. The limited number of outcome events and relatively small patient population are also a limitation for this study.

## Conclusions

In conclusion, survival was worse in patients with interval breast cancer than in those with screening-detected breast cancer. Interval cancer had more-aggressive histopathological characteristics, was more often associated with nodal and distant metastases, and received more chemotherapy and hormonal treatments than screening-detected cancer. Most breast cancers occurred in low-density breasts, and the number of extremely dense breasts was low in our study population. Our results indicate that better auditing and control of the regional screening program are required.

## Data Availability

The data that support the findings of this study are available upon request from the author [MS]. The data are not publicly available because they contain information that could compromise the privacy/consent of the research participants.

## Abbreviations

BMI: Body mass index
HER2: Human epidermal growth factor receptor 2
PACS: Picture archiving and communication system
BI-RADS: Breast Imaging-Reporting and Data System
SGR: Specific growth rate
DT: Doubling time
ER: Estrogen receptor
PR: Progesterone receptor
DCIS: Ductal carcinoma in situ
DFS: Disease-free survival
OS: Overall survival
DSS: Disease-specific survival
